# Association between psoriasis and lung cancer: two-sample Mendelian randomization analyses

**DOI:** 10.1186/s12890-022-02297-0

**Published:** 2023-01-05

**Authors:** Xiuqing Wang, Xiulan Wang, Hongkang Wang, Mingxing Yang, Wen Dong, Dan Shao

**Affiliations:** 1 Department of Respiratory Medicine, Hainan Cancer Hospital, Haikou, 570311 Hainan Province China; 2 Department of Respiratory Medicine, Hainan Provincial Hospital of Chinese Medicine, Haikou, 570203 Hainan Province China; 3 Department of Respiratory and Critical Care Medicine, Inner Mongolia Xing’an Meng People’s Hospital, Ulanhot, 137400 China

**Keywords:** Lung cancer, Psoriasis, Mendelian randomization, Smoking, Genetics

## Abstract

**Background:**

Observational studies reported an association between psoriasis and risk of lung cancer. However, whether psoriasis is causally associated with lung cancer is unclear.

**Methods:**

Genetic summary data of psoriasis were retrieved from two independent genome-wide association studies (GWAS). Genetic information of lung cancer was retrieved from GWAS of International Lung Cancer Consortium. A set of quality control steps were conducted to select instrumental tools. We performed two independent two-sample Mendelian randomization (MR) analyses and a meta-analysis based on the two independent MR estimates to assess the causal relationship between psoriasis and lung cancer (LUCA) as well as its subtypes, squamous cell carcinoma (LUSC) and adenocarcinoma (LUAD).

**Results:**

Between-SNP heterogeneity was present for most MR analyses, whereas horizontal pleiotropy was not detected for all MR analyses. Multiplicative random-effect inverse variance weighted (IVW-MRE) method was therefore selected as the primary MR approach. Both IVW-MRE estimates from the two independent MR analyses suggested that there was no significant causal relationship between psoriasis and LUCA as well as its histological subtypes. Sensitivity analyses using other four MR methods gave similar results. Meta-analysis of the two IVW-MRE derived MR estimates yielded an odds ratio (OR) of 1.00 (95% CI 0.95–1.06) for LUCA, 1.01 (95% CI 0.93–1.08) for LUSC, and 0.97 (95% CI 0.90–1.06) for LUAD.

**Conclusion:**

Our results do not support a genetic association between psoriasis and lung cancer and its subtypes. More population-based and experimental studies are warranted to further dissect the complex correlation between psoriasis and lung cancer.

**Supplementary Information:**

The online version contains supplementary material available at 10.1186/s12890-022-02297-0.

## Background

Lung cancer (LUCA), mainly including squamous cell carcinoma (LUSC) and adenocarcinoma (LUAD), is the second most commonly diagnosed malignancy and the leading cause of cancer death in 2020 worldwide, representing approximately 11.4% (2.2 million) cancers diagnosed and 18.0% (1.8 million) deaths [1]. LUCA involves a set of risk factors, including smoking, air pollution, cooking oil fume, obesity, and genetic variants [2–5]. Moreover, previous epidemiological studies reported that some immune-mediated diseases were associated with an increased risk of LUCA [6–10]. For instance, based on the UK Biobank cohort, He et al. found that psoriasis conferred an approximately 60% increased risk on LUCA among Europeans [9]. Evidence from a meta-analysis also suggested that psoriasis patients had a 1.26-fold increased risk of LUCA compared with those free of psoriasis [7]. These findings indicate that psoriasis might play a role in the development of LUCA. However, owing to the inherent limitations of observational studies, such as information biases, confounders, and reverse causality, the observed association between psoriasis and LUCA may be subject to a chance finding and therefore warrants further validation.

Mendelian randomization (MR) is an analogue of randomized controlled trial and leverages genetic information as instrumental variable for exposure. Due to the allocation of allele is occurred during meiosis and is independent of environmental exposures, MR analysis was deemed to be less susceptible to underlying confounders and enabled to infer causal relationship when the statistical assumptions were met [11, 12]. So far, MR analysis has been widely used to explore the associations between exposures and outcomes and serves as a good complement for observational studies [13–16].

There were a hundred of MR analyses for LUCA have been performed and published [17–19]. For example, MR estimates from Larsson et al. supported the well-established relationship between smoking and LUCA, highlighting the importance of smoking cessation for LUCA prevention [20]. Another MR study from Dimitrakopoulou et al. found that there is little evidence for a causal association between circulating vitamin D concentration and the risk of LUCA, and suggested that population-wide screening for vitamin D deficiency and subsequent widespread vitamin D supplementation should not be recommended as a strategy for primary cancer prevention [21]. Hence, MR study has significance for LUCA prevention and to understand LUCA pathogenesis. However, there was yet no MR analysis has been performed to assess the association between psoriasis and LUCA. To fill this gap, in the current study, we performed two-sample MR analyses to assess the potentially causal relationship between psoriasis and LUCA.

## Methods

### Study design

**Fig. 1:**
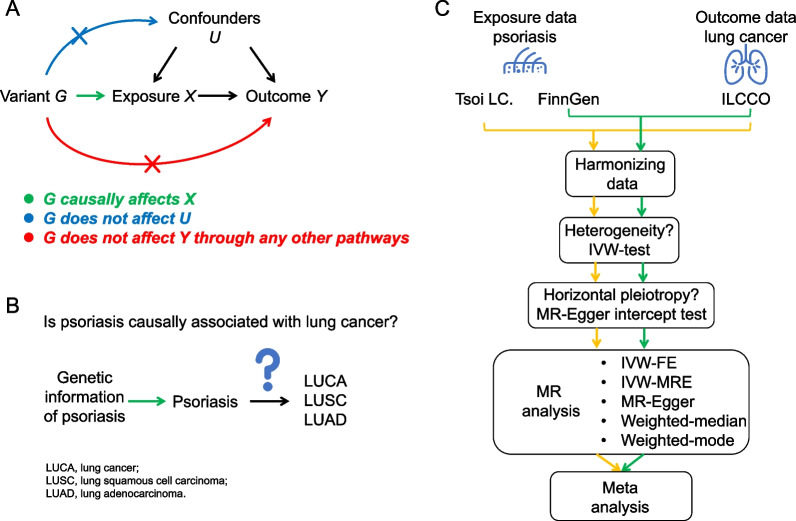
The schematic representations of our study. **A** the basic schema of Mendelian randomization (MR) analysis; **B** the study design of our MR analysis; **C** the flow chart of our MR analysis. ILCCO, international lung cancer consortium; IVW-FE, fixed-effect inverse variance weighted model; IVW-MRE, multiplicative random-effect inverse variance weighted model

The basic schema of MR analysis is shown in Fig. 1A [22]. In the current study, we set psoriasis and LUCA as exposure and outcome, respectively (Fig. 1B). The genetic information of psoriasis that derived from genome-wide association study (GWAS) were set as instrumental variable (Fig. 1B).

### Psoriasis GWAS

For psoriasis, we retrieved the GWAS summary data (coefficient and standard error for each SNP) from Tsoi LC et al. (PMID: 28,537,254) [23] and FinnGen (https://www.finngen.fi/en) via GWAS Catalog (inquiry code: GCST004346) and IEU-OpenGWAS project (inquiry code: finn-b-L12_PSORIASIS) online platform, respectively. In the GWAS of Tsoi LC et al., the authors included totally 19 032 psoriasis cases and 286 769 controls who were European ancestry from 8 independent cohorts, and performed a meta-analysis of 9 113 515 markers with good imputation quality in at least four data sets, using the inverse-variance approach [23]. A total of 63 loci were identified and could explain nearly 28% of the genetic heritability.

FinnGen study was launched in Finland in 2017 and is a population-based study that combines genome information with digital health care data. FinnGen individuals were genotyped with Illumina and Affymetrix chip arrays (Illumina Inc., San Diego, and Thermo Fisher Scientific, Santa Clara, CA, USA). Chip genotype data were imputed using the population-specific SISu v3 imputation reference panel of 3775 whole genomes, yielding totally 16 962 023 variants. In the quality control process, participants with ambiguous gender, high genotype missingness (> 5%), excess heterozygosity and non-Finnish ancestry were excluded. Variants with high missingness (> 2%), low Hardy–Weinberg Equilibrium *P*-value (< 10^–6^) and minor allele count < 3 were excluded. Finally, 4510 psoriasis cases that identified by ICD-10 code L40 and 212 242 healthy controls were included for GWAS.

### Lung cancer GWAS

For LUCA and its subtypes LUSC and LUAD, we retrieved the GWAS summary data from Wang et al. [24] via IEU-OpenGWAS online platform using inquiry code of “ieu-a-966”, “ieu-a-967”, and “ieu-a-965”, respectively. In this GWAS, Wang et al. on behalf of the International Lung Cancer Consortium (ILCCO) performed a meta-analysis using inverse-variance approaches based on data from four previously reported lung cancer GWAS of European populations: the MDACC GWAS, the ICR GWAS, the NCI GWAS, and the IARC GWAS. A total of 11 348 LUCA cases (3275 LUSC and 3442 LUAD) and 15 861 controls were included. In the quality control process, individuals with low call rate (< 90%) and extremely high or low heterozygosity (*P* < 1.0 × 10^−4^), as well as non-European ancestry were excluded. SNPs with a RSQR < 0.30 with MaCH or an information measure Is < 0.40 with IMPUTE2 were deemed as poorly imputed and were excluded from the analyses. Nearly 9 million SNPs were finally included in GWAS.

### Selection of instrumental tools

We performed a set of quality control steps to select suitable genetic instrumental tools [13]. First, we extracted SNPs associated with psoriasis at the genome-wide significance level (*P* < 5 × 10^−8^). Second, to ensure the independence of genetic variables, we performed a clumping process (threshold of R^2^ was < 0.001, window size = 10 000 kb) using linkage disequilibrium (LD) estimates calculated from Europeans in 1000 Genomes project. Among those pairs of SNPs that had LD R^2^ above the specified threshold (i.e., 0.001), we removed the SNP with the relatively higher *P* value. SNPs absent from the LD reference panel were also removed. Third, SNPs with a minor allele frequency < 1% were excluded. We then extracted the GWAS summary data of the selected SNPs from outcome datasets. For SNP that was absent in the outcome GWAS, we used SNP that had a LD R^2^ > 0.8 as a proxy. Furthermore, ambiguous SNPs with inconcordant alleles and palindromic SNPs with an ambiguous strand were either directly excluded or corrected in MR analysis.

To detect the underlying weak instrumental variable bias, we calculated the F-statistic using following formula: *F* = *R*^*2*^*(n − k − 1)/k(1 − R*^*2*^*)*, where *R*^*2*^, *n*, and *k* denotes the proportion of variance of exposure explained by selected genetic tools, sample size of exposure GWAS, and number of selected genetic tools, respectively. A mean F-statistic > 10 suggests suitable instrumental variables [25].

### Mendelian randomization analysis

We performed two independent two-sample MR analyses in this study. The flow chart is shown in Fig. 1C. First, we harmonized the exposure data and outcome data by matching the SNPs. Second, we applied inverse-variance weighted (IVW) method to test between-SNP heterogeneity. *P* value of the Q-statistic > 0.05 means the absence of heterogeneity. Third, we used MR-Egger regression intercept test to identify the horizontal pleiotropy. Fourth, we chose primary MR method according to the testing of between-SNP heterogeneity and horizontal pleiotropy. Briefly, if there was neither heterogeneity nor pleiotropy, use fixed-effect IVW (IVW-FE); if there was heterogeneity but no pleiotropy, use multiplicative random-effect IVW (IVW-MRE); if there was pleiotropy with or without heterogeneity, use MR-Egger regression [26]. We applied five methods in the current study to ensure the robustness of results. Finally, we used fixed-effect model to meta-analyze the MR estimates based on the GWAS of Tsoi LC and FinnGen. Leave-one-out analysis was also performed to identify the influential SNPs. We used mRnd website to calculate the statistical power for MR analysis [27]. All statistics were performed using R program (version 4.0.3). MR analysis was implemented using *TwoSampleMR* [28] and *gwasrapidd* [29] packages. Meta-analysis was implemented using *Metaan* package.

## Results

### Instrumental variables

A total of 55 and 10 SNPs were selected from psoriasis GWAS of Tsoi LC and FinnGen, respectively (Table 1). The coefficients and standard errors of each SNP for both exposure and outcome are shown in Additional file 1: Tables S1-S6. The mean F-statistics were both > 10. IVW test suggested that between-SNP heterogeneity was present for 5 of 6 MR analyses, whereas horizontal pleiotropy was not detected for all MR analyses (*P* value of MR-Egger regression intercept test > 0.05) (Table 1). We therefore chose IVW-MRE as the primary MR method. Of note, in the current scenario, most MR analyses were less powered (< 80%) to detect a weak odds ratio (OR) between 0.95 and 1.05. For MR analysis based on Tsoi LC, MR analysis was able to detect an OR > 1.1 or < 0.9 with an almost sufficient power (80%) (Table 1).

**Table 1:** Mendelian randomization analyses between psoriasis and lung cancer

Psoriasis	Lung cancer	No. of SNP	Mean F-statistics	Heterogeneity test^*a*^		Horizontal pleiotropy^*b*^			OR (95% CI)^*c*^	Statistical power for detecting OR in 0.95–1.05	Statistical power for detecting OR < 0.9 or > 1.1
				Q-statistics	*P* value	Egger-intercept	se	*P* value			
Tsoi LC	LUCA	55	444.3	93.9	< 0.001	−0.0048	0.0116	0.682	1.00 (0.94–1.07)	0.59	0.99
	LUSC	55	444.3	65.8	0.014	−0.0058	0.0152	0.705	1.00 (0.92–1.09)	0.28	0.77
	LUAD	55	444.3	88.4	< 0.001	−0.0018	0.0173	0.918	0.99 (0.90–1.09)	0.28	0.79
FinnGen	LUCA	10	220.0	17.2	0.046	0.0051	0.0330	0.881	1.01 (0.91–1.13)	0.25	0.70
	LUSC	10	220.0	13.4	0.143	0.0302	0.0424	0.496	1.02 (0.88–1.18)	0.13	0.37
	LUAD	10	220.0	21.8	0.009	−0.0701	0.0531	0.223	0.93 (0.77–1.13)	0.13	0.38

^a^ results were derived from IVW-test

^b^ results were derived from MR-Egger regression

^c^ results were derived from IVW-MRE models

*LUCA* lung cancer; *LUSC* lung squamous cell carcinoma; *LUAD* lung adenocarcinoma

*se* standard error; *OR* odds ratio; *CI* confidence interval

### Association between psoriasis and lung cancer

**Fig. 2:**
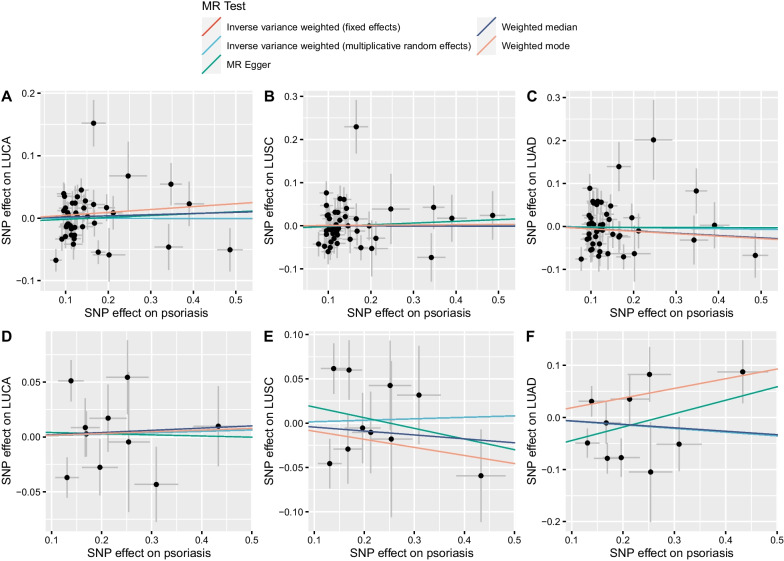
Scatter plots for MR analysis of the causal effect of psoriasis on lung cancer and its subtypes. **A** SNP effects on psoriasis and LUCA based on Tsoi et al. study; **B** SNP effects on psoriasis and LUSC based on Tsoi et al. study; **C** SNP effects on psoriasis and LUAD based on Tsoi et al. study; **D** SNP effects on psoriasis and LUCA based on FinnGen study; **E** SNP effects on psoriasis and LUSC based on FinnGen study; **F** SNP effects on psoriasis and LUAD based on FinnGen study. LUCA, lung cancer; LUSC, lung squamous cell carcinoma; LUAD, lung adenocarcinoma

**Fig. 3:**
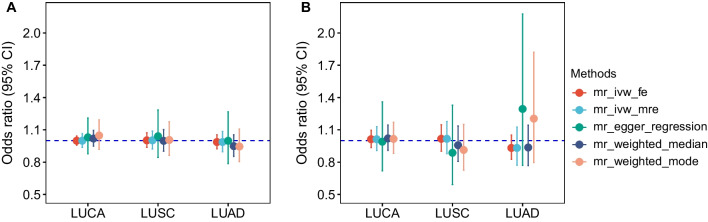
Estimates from different MR analysis of the causal effect of psoriasis on lung cancer and its subtypes. **A** estimates based on Tsoi et al. study; **B** estimates based on FinnGen study. LUCA, lung cancer; LUSC, lung squamous cell carcinoma; LUAD, lung adenocarcinoma

The scatter plots of six MR analyses are shown in Fig. 2. The MR estimates based on GWAS of Tsoi LC were much more consistent and robust than that based on GWAS of FinnGen. Based on psoriasis GWAS of Tsoi LC et al., IVW-MRE method suggested that there was no significant causal relationship between psoriasis and LUCA as well as its subtypes LUSC and LUAD (Table 1). The OR for LUCA, LUSC, and LUAD was 1.00 (95% CI 0.94–1.07), 1.00 (95% CI 0.92–1.09), and 0.99 (95% CI 0.90–1.09), respectively. Other four methods also reported similar results, albeit the nuances in MR estimates (Fig. 3A; Additional file 1: Table S7).

Likewise, based on psoriasis GWAS of FinnGen, IVW-MRE method did not detect significant causal relationship between psoriasis and LUCA and its subtypes (Table 1). The MR point estimates were close to 1. Other four methods did not report any conflicting result (Fig. 3B; Additional file 1: Table S7).

**Fig. 4:**
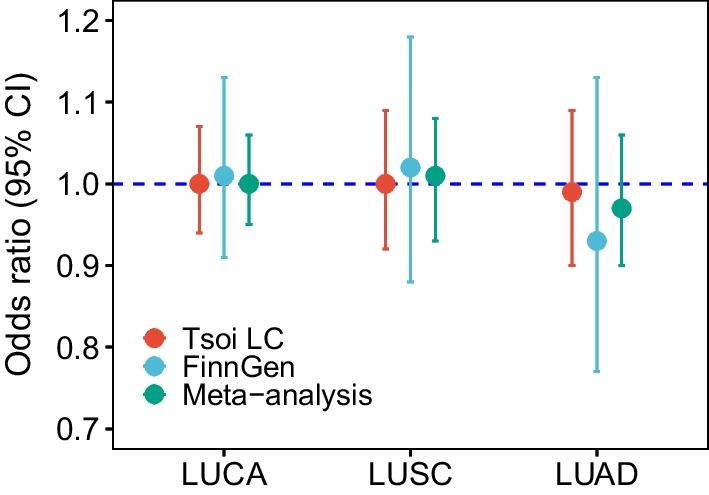
Meta-analysis estimates of MR results from multiplicative random-effect inverse variance weighted model based on GWAS of Tsoi LC et al. and FinnGen. LUCA, lung cancer; LUSC, lung squamous cell carcinoma; LUAD, lung adenocarcinoma

Meta-analysis of the two IVW-MRE derived MR estimates yielded an OR of 1.00 (95% CI 0.95–1.06) for LUCA, 1.01 (95% CI 0.93–1.08) for LUSC, and 0.97 (95% CI 0.90–1.06) for LUAD (Fig. 4). Leave-one-out analysis did not detect any influential SNP for all MR analyses (Additional file 2: Figures S1-S6).

## Discussion

In this MR study based on three large-scale GWAS, we found that psoriasis might not be causally associated with the risk of lung cancer as well as its histological subtypes, squamous cell carcinoma and adenocarcinoma. This finding was consistent in different GWAS data sources and was also validated in sensitivity analyses. Meta-analysis based on the two independent MR estimates yielded a concordant result.

Psoriasis is a common, chronic papulosquamous skin disease occurring worldwide and closely links to immune function [30]. The estimates of the prevalence of psoriasis in adults ranged from 0.51% to 11.43%, and in children from 0% to 1.37% [31]. The highest prevalence was in European populations [32]. A few of epidemiological studies reported that psoriasis was associated with an increased risk of lung cancer at different degrees [7, 9, 33–36]. The association was concordant in both sexes and in people with distinct ethnicities. For example, in the Iowa’s Women’s Health Study, the authors reported that psoriasis was associated with a 90% increased risk of lung cancer [37]. In a cohort study enrolled nearly 0.9 million Korean participants, psoriasis was found to be associated with a 14% and 20% increased risk of lung cancer in males and females, respectively [34]. Although cumulative evidences from observational studies support an association between psoriasis and lung cancer risk, the mechanisms underlying the observed relation are far from understood. Furthermore, whether the observational correlation connotes a causal relationship is still unclear. In this regard, MR analysis can provide more insights into the association of psoriasis with lung cancer.

In the current MR study, our results provide no genetic evidence for an association between psoriasis and lung cancer as well as its subtypes. This finding suggests that the observed association from population-based studies might be biased by underlying confounders and indicates that psoriasis may share common risk factors with lung cancer but itself is not a risk factor for lung cancer. For example, Prizment et al. found that the association between psoriasis and lung cancer became statistically non-significant when further adjusting for smoking, body mass index, education, physical activity, and hormone therapy use [37]. Chiesa F et al. found that the association between psoriasis and lung cancer was disappeared when limiting the study participants to non-smokers [35]. Moreover, in two large-scale epidemiological studies that had adjusted for smoking status of study participants, no significant association between psoriasis and lung cancer was detected [38, 39]. On the contrary, in several epidemiological studies reporting positive association between psoriasis and lung cancer, the common risk factors such as smoking were not adjusted for in the regression models [34, 36, 40]. Hence, in observational studies, it is not possible to totally tease out the role of confounding factors, particularly smoking, alcohol drinking, and obesity, which have all been reported to independently increase the risk of both lung cancer and psoriasis [18, 41–44].

The overlap between risk factors of these two conditions might partly explain the overrepresentation of lung cancer observed in patients with psoriasis. Cytokines that upregulated in psoriasis patients include TNF-α, IL-1β, IL-12, and IL-17A were also involved in the development of lung cancer [45–47], indicating that both psoriasis and lung cancer are closely involved with immune alteration [48]. In order to determine the true and pure impact of psoriasis on the risk of lung cancer, prospective studies with a careful consideration of the common risk factors especially smoking status should be conducted. However, MR analysis leveraging genetic information to some extent could serve as a good surrogate.

To our knowledge, this is the first MR analysis to assess the association between psoriasis and lung cancer. Our study has some strengths. First, compared to observational studies, our MR approach allows causal inference free from confounders and reverse causality. Second, the instrumental variables for psoriasis were derived from two large-scale GWAS that enrolled more than 220 thousand participants, thus ensuring the suitability of genetic tools. Third, our MR findings were consistent in subtypes of lung cancer and were validated by sensitivity analyses and meta-analysis. Although these notable advantages, the limitations of our study should also be noted here. First, the genetic data used in our analyses were from Europeans, thus limiting the extrapolation to other populations. Second, our MR analysis may be short in statistical power to detect a weak association between psoriasis and lung cancer. GWAS with more participants for lung cancer was therefore warranted in the future. Third, our estimates might also subject to the inherent pitfalls of MR analysis such as selection bias [49]. For example, the medication and treatment for psoriasis were not been considered in the GWAS, and might influence the selection of genetic tools used in our MR analysis.

## Conclusion

In conclusion, our MR analyses suggested there was no causal relationship between psoriasis and lung cancer. The observed association between these two diseases might be confounded by the shared risk factors such as smoking. More population-based and experimental investigations are warranted to further dissect the complex relation between psoriasis and lung cancer in the future. Although genetic evidence did not support the causal relationship, we should still strengthen the management of psoriasis patients, such as cancer screening and smoking cessation, to reduce the odds of lung cancer.

## Supplementary Information

**Additional file 1. Table S1** Genetic instrumental tools used in Mendelian randomization analysis of psoriasis with lung cancer based on GWAS of Tsoi LC. **Table S2** Genetic instrumental tools used in Mendelian randomization analysis of psoriasis with squamous cell lung cancer based on GWAS of Tsoi LC. **Table S3** Genetic instrumental tools used in Mendelian randomization analysis of psoriasis with lung adenocarcinoma based on GWAS of Tsoi LC. **Table S4** Genetic instrumental tools used in Mendelian randomization analysis of psoriasis with lung cancer based on GWAS of FinnGen. **Table S5** Genetic instrumental tools used in Mendelian randomization analysis of psoriasis with squamous cell lung cancer based on GWAS of FinnGen. **Table S6** Genetic instrumental tools used in Mendelian randomization analysis of psoriasis with lung adenocarcinoma based on GWAS of FinnGen. **Table S7** Results of Mendelian randomization on psoriasis and lung cancer.

**Additional file 2. Figure S1** Leave-one-out analysis for lung cancer based on GWAS of Tsoi LC. **Figure S2** Leave-one-out analysis for squamous cell lung cancer based on GWAS of Tsoi LC. **Figure S3** Leave-one-out analysis for lung adenocarcinoma based on GWAS of Tsoi LC. **Figure S4** Leave-one-out analysis for lung cancer based on GWAS of FinnGen. **Figure S5** Leave-one-out analysis for squamous cell lung cancer based on GWAS of FinnGen. **Figure S6** Leave-one-out analysis for lung adenocarcinoma based on GWAS of FinnGen.

## Data Availability

The GWAS data of psoriasis were retrieved from GWAS Catalog (https://www.ebi.ac.uk/gwas/studies/GCST004346) and IEU-OpenGWAS project (https://gwas.mrcieu.ac.uk/datasets/finn-b-L12_PSORIASIS/) online platform, respectively. The GWAS data of lung cancer were retrieved from IEU-OpenGWAS project (https://gwas.mrcieu.ac.uk/datasets/ieu-a-965/, https://gwas.mrcieu.ac.uk/datasets/ieu-a-966/, and https://gwas.mrcieu.ac.uk/datasets/ieu-a-967/).

## Abbreviations

MR: Mendelian randomization
LUCA: Lung cancer
LUSC: Lung squamous cell carcinoma
LUAD: Lung adenocarcinoma
IVW-FE: Fixed-effect inverse variance weighted model
IVW-MRE: Multiplicative random-effect inverse variance weighted model
GWAS: Genome-wide association study
