# Supplementary material for: Association between psoriasis and lung cancer: two-sample Mendelian randomization analyses
Source: BMC Pulm Med. 2023 Jan 5;23:4. doi: 10.1186/s12890-022-02297-0 (PMC9814449; doi:10.1186/s12890-022-02297-0)
Supplement: Supplementary file 1 — Additional file 1. Table S1 Genetic instrumental tools used in Mendelian randomization analysis of psoriasis with lung cancer based on GWAS of Tsoi LC. Table S2 Genetic instrumental tools used in Mendelian randomization analysis of psoriasis with squamous cell lung cancer based on GWAS of Tsoi LC. Table S3 Genetic instrumental tools used in Mendelian randomization analysis of psoriasis with lung adenocarcinoma based on GWAS of Tsoi LC. Table S4 Genetic instrumental tools used in Mendelian randomization analysis of psoriasis with lung cancer based on GWAS of FinnGen. Table S5 Genetic instrumental tools used in Mendelian randomization analysis of psoriasis with squamous cell lung cancer based on GWAS of FinnGen. Table S6 Genetic instrumental tools used in Mendelian randomization analysis of psoriasis with lung adenocarcinoma based on GWAS of FinnGen. Table S7 Results of Mendelian randomization on psoriasis and lung cancer. [file 12890_2022_2297_MOESM1_ESM.docx]

SUPPLEMENTARY TABLES

**Association between psoriasis and lung cancer: two-sample Mendelian randomization analyses**

Table S1. Genetic instrumental tools used in Mendelian randomization analysis on psoriasis and lung cancer, based on Tsoi LC et al.

| SNP | Effect allele | Other allele | CHR | Position | Exposure | | | Outcome | | |
| --- | --- | --- | --- | --- | --- | --- | --- | --- | --- | --- |
|  |  |  |  |  | beta | se | P value | beta | se | P value |
| rs10794648 | T | C | 1 | 24518206 | -0.1512 | 0.0186 | 5.00E-16 | -0.0025 | 0.0206 | 9.04E-01 |
| rs11053802 | T | C | 12 | 10597207 | 0.1044 | 0.0177 | 4.00E-09 | -0.0097 | 0.0194 | 6.22E-01 |
| rs11059675 | A | G | 12 | 122668326 | 0.0953 | 0.0170 | 2.00E-08 | 0.0011 | 0.0184 | 9.52E-01 |
| rs11065979 | T | C | 12 | 112059557 | 0.0770 | 0.0137 | 2.00E-08 | -0.0674 | 0.0179 | 5.65E-04 |
| rs1108618 | A | G | 10 | 81043743 | 0.1062 | 0.0158 | 2.00E-11 | 0.0054 | 0.0186 | 7.76E-01 |
| rs113935720 | T | C | 1 | 67713346 | 0.3904 | 0.0347 | 2.00E-29 | 0.0229 | 0.0354 | 5.42E-01 |
| rs11767350 | A | G | 7 | 37385365 | 0.0995 | 0.0156 | 2.00E-10 | 0.0163 | 0.0180 | 3.83E-01 |
| rs11795343 | T | G | 9 | 32523737 | 0.0918 | 0.0155 | 3.00E-09 | -0.0049 | 0.0180 | 7.90E-01 |
| rs12118303 | C | T | 1 | 172675097 | 0.1133 | 0.0180 | 3.00E-10 | -0.0172 | 0.0231 | 4.76E-01 |
| rs12188300 | A | T | 5 | 158829527 | -0.5294 | 0.0272 | 3.00E-84 | -0.0098 | 0.0364 | 7.93E-01 |
| rs1295685 | A | G | 5 | 131996445 | -0.1960 | 0.0198 | 5.00E-23 | -0.0168 | 0.0213 | 4.49E-01 |
| rs13080782 | A | G | 3 | 16996623 | -0.1198 | 0.0157 | 2.00E-14 | 0.0329 | 0.0174 | 7.31E-02 |
| rs144098432 | T | C | 18 | 51816394 | 0.1214 | 0.0170 | 1.00E-12 | -0.0148 | 0.0197 | 4.68E-01 |
| rs1707602 | T | G | 3 | 101647309 | -0.1061 | 0.0163 | 8.00E-11 | 0.0092 | 0.0189 | 6.29E-01 |
| rs17812953 | T | C | 21 | 36488822 | -0.1181 | 0.0218 | 6.00E-08 | 0.0416 | 0.0244 | 1.11E-01 |
| rs2145623 | C | G | 14 | 35839236 | 0.1521 | 0.0169 | 2.00E-19 | -0.0114 | 0.0199 | 5.79E-01 |
| rs2304856 | T | C | 17 | 78175483 | 0.1015 | 0.0177 | 1.00E-08 | -0.0138 | 0.0177 | 4.51E-01 |
| rs2451258 | T | C | 6 | 159506600 | -0.1109 | 0.0161 | 6.00E-12 | 0.0121 | 0.0188 | 5.33E-01 |
| rs2459446 | T | C | 10 | 75601596 | -0.1270 | 0.0160 | 2.00E-15 | -0.0028 | 0.0180 | 8.80E-01 |
| rs27044 | C | G | 5 | 96118852 | -0.1444 | 0.0166 | 4.00E-18 | -0.0227 | 0.0202 | 2.59E-01 |
| rs28624578 | T | C | 15 | 31637666 | 0.1655 | 0.0270 | 9.00E-10 | 0.0219 | 0.0232 | 3.66E-01 |
| rs28998802 | A | G | 17 | 26124908 | 0.2118 | 0.0216 | 9.00E-23 | 0.0086 | 0.0267 | 7.53E-01 |
| rs2944542 | G | C | 10 | 64375350 | 0.0770 | 0.0137 | 2.00E-08 | -0.0352 | 0.0191 | 6.07E-02 |
| rs34517439 | A | C | 1 | 78450517 | 0.1655 | 0.0281 | 4.00E-09 | 0.1519 | 0.0370 | 3.86E-06 |
| rs34536443 | C | G | 19 | 10463118 | -0.6762 | 0.0490 | 3.00E-43 | 0.1914 | 0.0582 | 1.50E-04 |
| rs35194171 | A | T | 2 | 61075209 | 0.1665 | 0.0157 | 2.00E-26 | 0.0195 | 0.0178 | 2.89E-01 |
| rs3900909 | C | G | 1 | 197757846 | -0.1005 | 0.0198 | 4.00E-07 | -0.0345 | 0.0239 | 1.44E-01 |
| rs41298997 | T | C | 1 | 206655331 | 0.1222 | 0.0218 | 2.00E-08 | 0.0241 | 0.0272 | 3.77E-01 |
| rs413024 | A | G | 16 | 11354091 | 0.1367 | 0.0170 | 1.00E-15 | 0.0450 | 0.0186 | 2.34E-02 |
| rs4561177 | A | G | 11 | 109962432 | 0.1239 | 0.0154 | 9.00E-16 | 0.0083 | 0.0180 | 6.53E-01 |
| rs4672505 | A | G | 2 | 62560332 | 0.1157 | 0.0159 | 3.00E-13 | 0.0240 | 0.0189 | 2.02E-01 |
| rs4804528 | T | G | 19 | 10886206 | -0.0994 | 0.0156 | 2.00E-10 | 0.0294 | 0.0186 | 1.09E-01 |
| rs4845453 | C | G | 1 | 152591953 | 0.1771 | 0.0163 | 2.00E-27 | -0.0055 | 0.0185 | 7.68E-01 |
| rs492602 | G | A | 19 | 49206417 | 0.1044 | 0.0145 | 7.00E-13 | 0.0087 | 0.0179 | 6.34E-01 |
| rs4942358 | A | C | 13 | 45321731 | -0.0972 | 0.0170 | 1.00E-08 | -0.0349 | 0.0203 | 7.98E-02 |
| rs55823223 | A | G | 17 | 73890363 | 0.1398 | 0.0244 | 1.00E-08 | -0.0137 | 0.0255 | 6.06E-01 |
| rs559406 | G | T | 18 | 12857002 | 0.0953 | 0.0147 | 1.00E-10 | 0.0120 | 0.0182 | 5.13E-01 |
| rs57137641 | A | G | 12 | 56741228 | -0.3421 | 0.0330 | 4.00E-25 | 0.0462 | 0.0368 | 2.05E-01 |
| rs5754387 | C | G | 22 | 21974703 | 0.1377 | 0.0192 | 7.00E-13 | -0.0356 | 0.0221 | 1.29E-01 |
| rs582757 | T | C | 6 | 138197824 | -0.1765 | 0.0168 | 1.00E-25 | 0.0543 | 0.0189 | 7.48E-03 |
| rs6067284 | A | G | 20 | 48574454 | -0.1462 | 0.0156 | 7.00E-21 | -0.0276 | 0.0178 | 1.38E-01 |
| rs61871342 | G | A | 10 | 102038641 | 0.0953 | 0.0159 | 2.00E-09 | 0.0393 | 0.0177 | 3.62E-02 |
| rs61907765 | T | C | 11 | 128391937 | 0.1426 | 0.0184 | 9.00E-15 | 0.0162 | 0.0216 | 4.56E-01 |
| rs6672420 | A | T | 1 | 25293201 | -0.1407 | 0.0157 | 3.00E-19 | -0.0450 | 0.0187 | 1.36E-02 |
| rs7184567 | T | C | 16 | 31021078 | 0.1275 | 0.0159 | 1.00E-15 | 0.0342 | 0.0192 | 6.99E-02 |
| rs73183592 | A | G | 13 | 40745693 | -0.2019 | 0.0379 | 1.00E-07 | 0.0588 | 0.0428 | 1.62E-01 |
| rs74817271 | A | G | 5 | 150469973 | 0.4863 | 0.0301 | 1.00E-58 | -0.0506 | 0.0345 | 1.79E-01 |
| rs7524364 | A | G | 1 | 8286009 | 0.1208 | 0.0193 | 4.00E-10 | -0.0273 | 0.0223 | 2.45E-01 |
| rs76959677 | G | A | 10 | 89824771 | 0.2469 | 0.0445 | 3.00E-08 | 0.0675 | 0.0546 | 2.09E-01 |
| rs7748720 | A | G | 6 | 20689945 | 0.1676 | 0.0188 | 6.00E-19 | -0.0080 | 0.0212 | 7.10E-01 |
| rs8070763 | T | C | 17 | 40536396 | -0.1024 | 0.0161 | 2.00E-10 | 0.0120 | 0.0190 | 5.42E-01 |
| rs887314 | T | G | 11 | 64053157 | 0.0953 | 0.0166 | 1.00E-08 | 0.0016 | 0.0193 | 9.34E-01 |
| rs9481169 | T | G | 6 | 111929862 | 0.3475 | 0.0255 | 2.00E-42 | 0.0543 | 0.0336 | 9.87E-02 |
| rs9504361 | A | G | 6 | 577820 | 0.0910 | 0.0156 | 5.00E-09 | -0.0335 | 0.0184 | 6.34E-02 |
| rs9513593 | G | A | 13 | 99950260 | 0.1133 | 0.0206 | 4.00E-08 | -0.0270 | 0.0224 | 2.52E-01 |

Table S2. Genetic instrumental tools used in Mendelian randomization analysis on psoriasis and squamous cell lung cancer, based on Tsoi LC et al.

| SNP | Effect allele | Other allele | CHR | Position | Exposure | | | Outcome | | |
| --- | --- | --- | --- | --- | --- | --- | --- | --- | --- | --- |
|  |  |  |  |  | beta | se | P value | beta | se | P value |
| rs10794648 | T | C | 1 | 24518206 | -0.1512 | 0.0186 | 5.00E-16 | 0.0314 | 0.0323 | 3.31E-01 |
| rs11053802 | T | C | 12 | 10597207 | 0.1044 | 0.0177 | 4.00E-09 | -0.0033 | 0.0297 | 9.13E-01 |
| rs11059675 | A | G | 12 | 122668326 | 0.0953 | 0.0170 | 2.00E-08 | -0.0201 | 0.0276 | 4.88E-01 |
| rs11065979 | T | C | 12 | 112059557 | 0.0770 | 0.0137 | 2.00E-08 | -0.0424 | 0.0281 | 1.60E-01 |
| rs1108618 | A | G | 10 | 81043743 | 0.1062 | 0.0158 | 2.00E-11 | -0.0377 | 0.0297 | 2.00E-01 |
| rs113935720 | T | C | 1 | 67713346 | 0.3904 | 0.0347 | 2.00E-29 | 0.0173 | 0.0543 | 7.67E-01 |
| rs11767350 | A | G | 7 | 37385365 | 0.0995 | 0.0156 | 2.00E-10 | 0.0251 | 0.0274 | 3.86E-01 |
| rs11795343 | T | G | 9 | 32523737 | 0.0918 | 0.0155 | 3.00E-09 | -0.0187 | 0.0273 | 5.13E-01 |
| rs12118303 | C | T | 1 | 172675097 | 0.1133 | 0.0180 | 3.00E-10 | -0.0297 | 0.0350 | 4.26E-01 |
| rs12188300 | A | T | 5 | 158829527 | -0.5294 | 0.0272 | 3.00E-84 | 0.0593 | 0.0522 | 3.12E-01 |
| rs1295685 | A | G | 5 | 131996445 | -0.1960 | 0.0198 | 5.00E-23 | 0.0006 | 0.0329 | 9.86E-01 |
| rs13080782 | A | G | 3 | 16996623 | -0.1198 | 0.0157 | 2.00E-14 | 0.0124 | 0.0273 | 6.62E-01 |
| rs144098432 | T | C | 18 | 51816394 | 0.1214 | 0.0170 | 1.00E-12 | -0.0212 | 0.0299 | 5.00E-01 |
| rs1707602 | T | G | 3 | 101647309 | -0.1061 | 0.0163 | 8.00E-11 | 0.0095 | 0.0291 | 7.48E-01 |
| rs17812953 | T | C | 21 | 36488822 | -0.1181 | 0.0218 | 6.00E-08 | -0.0023 | 0.0391 | 9.55E-01 |
| rs2145623 | C | G | 14 | 35839236 | 0.1521 | 0.0169 | 2.00E-19 | -0.0189 | 0.0304 | 5.54E-01 |
| rs2304856 | T | C | 17 | 78175483 | 0.1015 | 0.0177 | 1.00E-08 | -0.0074 | 0.0273 | 7.95E-01 |
| rs2451258 | T | C | 6 | 159506600 | -0.1109 | 0.0161 | 6.00E-12 | 0.0185 | 0.0288 | 5.40E-01 |
| rs2459446 | T | C | 10 | 75601596 | -0.1270 | 0.0160 | 2.00E-15 | -0.0127 | 0.0272 | 6.53E-01 |
| rs27044 | C | G | 5 | 96118852 | -0.1444 | 0.0166 | 4.00E-18 | 0.0076 | 0.0301 | 8.08E-01 |
| rs28624578 | T | C | 15 | 31637666 | 0.1655 | 0.0270 | 9.00E-10 | 0.0159 | 0.0355 | 6.71E-01 |
| rs28998802 | A | G | 17 | 26124908 | 0.2118 | 0.0216 | 9.00E-23 | -0.0292 | 0.0391 | 4.86E-01 |
| rs2944542 | G | C | 10 | 64375350 | 0.0770 | 0.0137 | 2.00E-08 | -0.0019 | 0.0284 | 9.49E-01 |
| rs34517439 | A | C | 1 | 78450517 | 0.1655 | 0.0281 | 4.00E-09 | 0.2295 | 0.0620 | 9.80E-06 |
| rs34536443 | C | G | 19 | 10463118 | -0.6762 | 0.0490 | 3.00E-43 | 0.1687 | 0.0856 | 3.06E-02 |
| rs35194171 | A | T | 2 | 61075209 | 0.1665 | 0.0157 | 2.00E-26 | 0.0266 | 0.0269 | 3.49E-01 |
| rs3900909 | C | G | 1 | 197757846 | -0.1005 | 0.0198 | 4.00E-07 | 0.0025 | 0.0358 | 9.46E-01 |
| rs41298997 | T | C | 1 | 206655331 | 0.1222 | 0.0218 | 2.00E-08 | 0.0309 | 0.0417 | 4.64E-01 |
| rs413024 | A | G | 16 | 11354091 | 0.1367 | 0.0170 | 1.00E-15 | 0.0610 | 0.0281 | 4.72E-02 |
| rs4561177 | A | G | 11 | 109962432 | 0.1239 | 0.0154 | 9.00E-16 | -0.0064 | 0.0279 | 8.22E-01 |
| rs4672505 | A | G | 2 | 62560332 | 0.1157 | 0.0159 | 3.00E-13 | 0.0313 | 0.0293 | 2.85E-01 |
| rs4804528 | T | G | 19 | 10886206 | -0.0994 | 0.0156 | 2.00E-10 | 0.0601 | 0.0294 | 3.45E-02 |
| rs4845453 | C | G | 1 | 152591953 | 0.1771 | 0.0163 | 2.00E-27 | -0.0149 | 0.0286 | 6.06E-01 |
| rs492602 | G | A | 19 | 49206417 | 0.1044 | 0.0145 | 7.00E-13 | -0.0506 | 0.0290 | 7.40E-02 |
| rs4942358 | A | C | 13 | 45321731 | -0.0972 | 0.0170 | 1.00E-08 | -0.0236 | 0.0307 | 4.46E-01 |
| rs55823223 | A | G | 17 | 73890363 | 0.1398 | 0.0244 | 1.00E-08 | 0.0210 | 0.0403 | 6.10E-01 |
| rs559406 | G | T | 18 | 12857002 | 0.0953 | 0.0147 | 1.00E-10 | 0.0470 | 0.0290 | 9.81E-02 |
| rs57137641 | A | G | 12 | 56741228 | -0.3421 | 0.0330 | 4.00E-25 | 0.0737 | 0.0562 | 1.81E-01 |
| rs5754387 | C | G | 22 | 21974703 | 0.1377 | 0.0192 | 7.00E-13 | 0.0036 | 0.0349 | 9.19E-01 |
| rs582757 | T | C | 6 | 138197824 | -0.1765 | 0.0168 | 1.00E-25 | 0.0510 | 0.0291 | 1.06E-01 |
| rs6067284 | A | G | 20 | 48574454 | -0.1462 | 0.0156 | 7.00E-21 | 0.0003 | 0.0281 | 9.91E-01 |
| rs61871342 | G | A | 10 | 102038641 | 0.0953 | 0.0159 | 2.00E-09 | 0.0762 | 0.0263 | 9.09E-03 |
| rs61907765 | T | C | 11 | 128391937 | 0.1426 | 0.0184 | 9.00E-15 | 0.0403 | 0.0338 | 2.29E-01 |
| rs6672420 | A | T | 1 | 25293201 | -0.1407 | 0.0157 | 3.00E-19 | -0.0285 | 0.0283 | 3.14E-01 |
| rs7184567 | T | C | 16 | 31021078 | 0.1275 | 0.0159 | 1.00E-15 | 0.0626 | 0.0304 | 3.29E-02 |
| rs73183592 | A | G | 13 | 40745693 | -0.2019 | 0.0379 | 1.00E-07 | 0.0528 | 0.0647 | 4.20E-01 |
| rs74817271 | A | G | 5 | 150469973 | 0.4863 | 0.0301 | 1.00E-58 | 0.0239 | 0.0560 | 6.79E-01 |
| rs7524364 | A | G | 1 | 8286009 | 0.1208 | 0.0193 | 4.00E-10 | -0.0412 | 0.0338 | 2.59E-01 |
| rs76959677 | G | A | 10 | 89824771 | 0.2469 | 0.0445 | 3.00E-08 | 0.0388 | 0.0814 | 6.48E-01 |
| rs7748720 | A | G | 6 | 20689945 | 0.1676 | 0.0188 | 6.00E-19 | -0.0125 | 0.0325 | 7.06E-01 |
| rs8070763 | T | C | 17 | 40536396 | -0.1024 | 0.0161 | 2.00E-10 | -0.0164 | 0.0300 | 5.88E-01 |
| rs887314 | T | G | 11 | 64053157 | 0.0953 | 0.0166 | 1.00E-08 | -0.0120 | 0.0299 | 6.95E-01 |
| rs9481169 | T | G | 6 | 111929862 | 0.3475 | 0.0255 | 2.00E-42 | 0.0426 | 0.0505 | 4.02E-01 |
| rs9504361 | A | G | 6 | 577820 | 0.0910 | 0.0156 | 5.00E-09 | -0.0477 | 0.0285 | 8.81E-02 |
| rs9513593 | G | A | 13 | 99950260 | 0.1133 | 0.0206 | 4.00E-08 | -0.0065 | 0.0348 | 8.57E-01 |

Table S3. Genetic instrumental tools used in Mendelian randomization analysis on psoriasis and lung adenocarcinoma, based on Tsoi LC et al.

| SNP | Effect allele | Other allele | CHR | Position | Exposure | | | Outcome | | |
| --- | --- | --- | --- | --- | --- | --- | --- | --- | --- | --- |
|  |  |  |  |  | beta | se | P value | beta | se | P value |
| rs10794648 | T | C | 1 | 24518206 | -0.1512 | 0.0186 | 5.00E-16 | 0.0183 | 0.0320 | 5.73E-01 |
| rs11053802 | T | C | 12 | 10597207 | 0.1044 | 0.0177 | 4.00E-09 | -0.0408 | 0.0309 | 1.81E-01 |
| rs11059675 | A | G | 12 | 122668326 | 0.0953 | 0.0170 | 2.00E-08 | 0.0051 | 0.0282 | 8.58E-01 |
| rs11065979 | T | C | 12 | 112059557 | 0.0770 | 0.0137 | 2.00E-08 | -0.0760 | 0.0275 | 1.30E-02 |
| rs1108618 | A | G | 10 | 81043743 | 0.1062 | 0.0158 | 2.00E-11 | 0.0545 | 0.0271 | 6.43E-02 |
| rs113935720 | T | C | 1 | 67713346 | 0.3904 | 0.0347 | 2.00E-29 | 0.0028 | 0.0535 | 9.61E-01 |
| rs11767350 | A | G | 7 | 37385365 | 0.0995 | 0.0156 | 2.00E-10 | 0.0198 | 0.0276 | 4.94E-01 |
| rs11795343 | T | G | 9 | 32523737 | 0.0918 | 0.0155 | 3.00E-09 | 0.0164 | 0.0282 | 5.65E-01 |
| rs12118303 | C | T | 1 | 172675097 | 0.1133 | 0.0180 | 3.00E-10 | 0.0510 | 0.0369 | 1.61E-01 |
| rs12188300 | A | T | 5 | 158829527 | -0.5294 | 0.0272 | 3.00E-84 | -0.0875 | 0.0604 | 1.35E-01 |
| rs1295685 | A | G | 5 | 131996445 | -0.1960 | 0.0198 | 5.00E-23 | -0.0203 | 0.0329 | 5.60E-01 |
| rs13080782 | A | G | 3 | 16996623 | -0.1198 | 0.0157 | 2.00E-14 | 0.0407 | 0.0265 | 1.52E-01 |
| rs144098432 | T | C | 18 | 51816394 | 0.1214 | 0.0170 | 1.00E-12 | -0.0065 | 0.0305 | 8.38E-01 |
| rs1707602 | T | G | 3 | 101647309 | -0.1061 | 0.0163 | 8.00E-11 | -0.0046 | 0.0286 | 8.77E-01 |
| rs17812953 | T | C | 21 | 36488822 | -0.1181 | 0.0218 | 6.00E-08 | 0.0693 | 0.0361 | 8.55E-02 |
| rs2145623 | C | G | 14 | 35839236 | 0.1521 | 0.0169 | 2.00E-19 | -0.0264 | 0.0302 | 4.09E-01 |
| rs2304856 | T | C | 17 | 78175483 | 0.1015 | 0.0177 | 1.00E-08 | 0.0084 | 0.0278 | 7.67E-01 |
| rs2451258 | T | C | 6 | 159506600 | -0.1109 | 0.0161 | 6.00E-12 | 0.0237 | 0.0284 | 4.29E-01 |
| rs2459446 | T | C | 10 | 75601596 | -0.1270 | 0.0160 | 2.00E-15 | 0.0087 | 0.0279 | 7.60E-01 |
| rs27044 | C | G | 5 | 96118852 | -0.1444 | 0.0166 | 4.00E-18 | -0.0466 | 0.0318 | 1.36E-01 |
| rs28624578 | T | C | 15 | 31637666 | 0.1655 | 0.0270 | 9.00E-10 | -0.0245 | 0.0369 | 5.12E-01 |
| rs28998802 | A | G | 17 | 26124908 | 0.2118 | 0.0216 | 9.00E-23 | -0.0105 | 0.0402 | 8.04E-01 |
| rs2944542 | G | C | 10 | 64375350 | 0.0770 | 0.0137 | 2.00E-08 | -0.0436 | 0.0294 | 1.32E-01 |
| rs34517439 | A | C | 1 | 78450517 | 0.1655 | 0.0281 | 4.00E-09 | 0.1394 | 0.0572 | 7.80E-03 |
| rs34536443 | C | G | 19 | 10463118 | -0.6762 | 0.0490 | 3.00E-43 | 0.3095 | 0.0995 | 8.65E-05 |
| rs35194171 | A | T | 2 | 61075209 | 0.1665 | 0.0157 | 2.00E-26 | -0.0227 | 0.0282 | 4.25E-01 |
| rs3900909 | C | G | 1 | 197757846 | -0.1005 | 0.0198 | 4.00E-07 | -0.0400 | 0.0365 | 2.71E-01 |
| rs41298997 | T | C | 1 | 206655331 | 0.1222 | 0.0218 | 2.00E-08 | 0.0030 | 0.0409 | 9.43E-01 |
| rs413024 | A | G | 16 | 11354091 | 0.1367 | 0.0170 | 1.00E-15 | 0.0283 | 0.0290 | 3.57E-01 |
| rs4561177 | A | G | 11 | 109962432 | 0.1239 | 0.0154 | 9.00E-16 | 0.0564 | 0.0264 | 4.95E-02 |
| rs4672505 | A | G | 2 | 62560332 | 0.1157 | 0.0159 | 3.00E-13 | 0.0585 | 0.0300 | 4.42E-02 |
| rs4804528 | T | G | 19 | 10886206 | -0.0994 | 0.0156 | 2.00E-10 | 0.0550 | 0.0291 | 5.22E-02 |
| rs4845453 | C | G | 1 | 152591953 | 0.1771 | 0.0163 | 2.00E-27 | -0.0152 | 0.0287 | 6.01E-01 |
| rs492602 | G | A | 19 | 49206417 | 0.1044 | 0.0145 | 7.00E-13 | 0.0584 | 0.0258 | 3.81E-02 |
| rs4942358 | A | C | 13 | 45321731 | -0.0972 | 0.0170 | 1.00E-08 | -0.0888 | 0.0329 | 4.20E-03 |
| rs55823223 | A | G | 17 | 73890363 | 0.1398 | 0.0244 | 1.00E-08 | -0.0635 | 0.0370 | 1.21E-01 |
| rs559406 | G | T | 18 | 12857002 | 0.0953 | 0.0147 | 1.00E-10 | -0.0276 | 0.0268 | 3.30E-01 |
| rs57137641 | A | G | 12 | 56741228 | -0.3421 | 0.0330 | 4.00E-25 | 0.0317 | 0.0567 | 5.85E-01 |
| rs5754387 | C | G | 22 | 21974703 | 0.1377 | 0.0192 | 7.00E-13 | -0.0863 | 0.0332 | 2.17E-02 |
| rs582757 | T | C | 6 | 138197824 | -0.1765 | 0.0168 | 1.00E-25 | 0.0708 | 0.0287 | 2.57E-02 |
| rs6067284 | A | G | 20 | 48574454 | -0.1462 | 0.0156 | 7.00E-21 | -0.0477 | 0.0268 | 9.91E-02 |
| rs61871342 | G | A | 10 | 102038641 | 0.0953 | 0.0159 | 2.00E-09 | 0.0258 | 0.0274 | 3.74E-01 |
| rs61907765 | T | C | 11 | 128391937 | 0.1426 | 0.0184 | 9.00E-15 | 0.0162 | 0.0331 | 6.30E-01 |
| rs6672420 | A | T | 1 | 25293201 | -0.1407 | 0.0157 | 3.00E-19 | -0.0113 | 0.0279 | 6.89E-01 |
| rs7184567 | T | C | 16 | 31021078 | 0.1275 | 0.0159 | 1.00E-15 | 0.0044 | 0.0288 | 8.81E-01 |
| rs73183592 | A | G | 13 | 40745693 | -0.2019 | 0.0379 | 1.00E-07 | 0.0634 | 0.0654 | 3.32E-01 |
| rs74817271 | A | G | 5 | 150469973 | 0.4863 | 0.0301 | 1.00E-58 | -0.0675 | 0.0518 | 2.50E-01 |
| rs7524364 | A | G | 1 | 8286009 | 0.1208 | 0.0193 | 4.00E-10 | -0.0588 | 0.0335 | 1.10E-01 |
| rs76959677 | G | A | 10 | 89824771 | 0.2469 | 0.0445 | 3.00E-08 | 0.2016 | 0.0928 | 1.41E-02 |
| rs7748720 | A | G | 6 | 20689945 | 0.1676 | 0.0188 | 6.00E-19 | -0.0216 | 0.0332 | 5.20E-01 |
| rs8070763 | T | C | 17 | 40536396 | -0.1024 | 0.0161 | 2.00E-10 | 0.0533 | 0.0281 | 8.11E-02 |
| rs887314 | T | G | 11 | 64053157 | 0.0953 | 0.0166 | 1.00E-08 | -0.0176 | 0.0304 | 5.67E-01 |
| rs9481169 | T | G | 6 | 111929862 | 0.3475 | 0.0255 | 2.00E-42 | 0.0826 | 0.0523 | 1.02E-01 |
| rs9504361 | A | G | 6 | 577820 | 0.0910 | 0.0156 | 5.00E-09 | -0.0298 | 0.0280 | 2.86E-01 |
| rs9513593 | G | A | 13 | 99950260 | 0.1133 | 0.0206 | 4.00E-08 | -0.0121 | 0.0347 | 7.39E-01 |

Table S4. Genetic instrumental tools used in Mendelian randomization analysis on psoriasis and lung cancer, based on FinnGen.

| SNP | Effect allele | Other allele | CHR | Position | Exposure | | | Outcome | | |
| --- | --- | --- | --- | --- | --- | --- | --- | --- | --- | --- |
|  |  |  |  |  | beta | se | P value | beta | se | P value |
| rs10829130 | A | G | 10 | 27174346 | 0.1965 | 0.0359 | 4.24E-08 | -0.0277 | 0.0255 | 3.04E-01 |
| rs12188300 | T | A | 5 | 158829527 | 0.4331 | 0.0495 | 2.24E-18 | 0.0098 | 0.0364 | 7.93E-01 |
| rs12713428 | C | A | 2 | 61118113 | 0.1694 | 0.0261 | 8.11E-11 | 0.0027 | 0.0207 | 8.96E-01 |
| rs138009430 | A | C | 16 | 27316975 | 0.2538 | 0.0423 | 1.94E-09 | -0.0046 | 0.0639 | 9.47E-01 |
| rs17728338 | A | G | 5 | 150478318 | 0.3092 | 0.0439 | 1.76E-12 | -0.0433 | 0.0342 | 2.42E-01 |
| rs2021511 | T | C | 16 | 11344903 | -0.1387 | 0.0254 | 4.75E-08 | -0.0511 | 0.0189 | 1.17E-02 |
| rs28998802 | A | G | 17 | 26124908 | 0.1672 | 0.0289 | 7.41E-09 | 0.0086 | 0.0267 | 7.53E-01 |
| rs60600003 | G | T | 7 | 37382465 | 0.2128 | 0.0372 | 1.03E-08 | 0.0171 | 0.0307 | 5.82E-01 |
| rs674451 | C | T | 6 | 138216788 | 0.1307 | 0.0235 | 2.82E-08 | -0.0370 | 0.0184 | 5.75E-02 |
| rs9481169 | T | G | 6 | 111929862 | 0.2515 | 0.0422 | 2.47E-09 | 0.0543 | 0.0336 | 9.87E-02 |

Table S5. Genetic instrumental tools used in Mendelian randomization analysis on psoriasis and squamous cell lung cancer, based on FinnGen.

| SNP | Effect allele | Other allele | CHR | Position | Exposure | | | Outcome | | |
| --- | --- | --- | --- | --- | --- | --- | --- | --- | --- | --- |
|  |  |  |  |  | beta | se | P value | beta | se | P value |
| rs10829130 | A | G | 10 | 27174346 | 0.1965 | 0.0359 | 4.24E-08 | -0.0054 | 0.0397 | 0.89679 |
| rs12188300 | T | A | 5 | 1.59E+08 | 0.4331 | 0.0495 | 2.24E-18 | -0.0593 | 0.0522 | 0.311786 |
| rs12713428 | C | A | 2 | 61118113 | 0.1694 | 0.0261 | 8.11E-11 | 0.0600 | 0.0335 | 0.065136 |
| rs138009430 | A | C | 16 | 27316975 | 0.2538 | 0.0423 | 1.94E-09 | -0.0179 | 0.0881 | 0.855799 |
| rs17728338 | A | G | 5 | 1.5E+08 | 0.3092 | 0.0439 | 1.76E-12 | 0.0318 | 0.0555 | 0.576301 |
| rs2021511 | T | C | 16 | 11344903 | -0.1387 | 0.0254 | 4.75E-08 | -0.0617 | 0.0286 | 0.049072 |
| rs28998802 | A | G | 17 | 26124908 | 0.1672 | 0.0289 | 7.41E-09 | -0.0292 | 0.0391 | 0.48551 |
| rs60600003 | G | T | 7 | 37382465 | 0.2128 | 0.0372 | 1.03E-08 | -0.0104 | 0.0460 | 0.831606 |
| rs674451 | C | T | 6 | 1.38E+08 | 0.1307 | 0.0235 | 2.82E-08 | -0.0456 | 0.0281 | 0.131488 |
| rs9481169 | T | G | 6 | 1.12E+08 | 0.2515 | 0.0422 | 2.47E-09 | 0.0426 | 0.0505 | 0.40197 |

Table S6. Genetic instrumental tools used in Mendelian randomization analysis on psoriasis and lung adenocarcinoma, based on FinnGen.

| SNP | Effect allele | Other allele | CHR | Position | Exposure | | | Outcome | | |
| --- | --- | --- | --- | --- | --- | --- | --- | --- | --- | --- |
|  |  |  |  |  | beta | se | P value | beta | se | P value |
| rs10829130 | A | G | 10 | 27174346 | 0.1965 | 0.0359 | 4.24E-08 | -0.0772 | 0.0367 | 6.18E-02 |
| rs12188300 | T | A | 5 | 158829527 | 0.4331 | 0.0495 | 2.24E-18 | 0.0875 | 0.0604 | 1.35E-01 |
| rs12713428 | C | A | 2 | 61118113 | 0.1694 | 0.0261 | 8.11E-11 | -0.0785 | 0.0293 | 1.63E-02 |
| rs138009430 | A | C | 16 | 27316975 | 0.2538 | 0.0423 | 1.94E-09 | -0.1047 | 0.0960 | 3.81E-01 |
| rs17728338 | A | G | 5 | 150478318 | 0.3092 | 0.0439 | 1.76E-12 | -0.0513 | 0.0519 | 3.75E-01 |
| rs2021511 | T | C | 16 | 11344903 | -0.1387 | 0.0254 | 4.75E-08 | -0.0309 | 0.0295 | 3.24E-01 |
| rs28998802 | A | G | 17 | 26124908 | 0.1672 | 0.0289 | 7.41E-09 | -0.0105 | 0.0402 | 8.04E-01 |
| rs60600003 | G | T | 7 | 37382465 | 0.2128 | 0.0372 | 1.03E-08 | 0.0351 | 0.0469 | 4.59E-01 |
| rs674451 | C | T | 6 | 138216788 | 0.1307 | 0.0235 | 2.82E-08 | -0.0491 | 0.0281 | 1.07E-01 |
| rs9481169 | T | G | 6 | 111929862 | 0.2515 | 0.0422 | 2.47E-09 | 0.0826 | 0.0523 | 1.02E-01 |

Table S7. Results of Mendelian randomization on psoriasis and lung cancer.

| Database | Methods | Disease | beta | se | P value | OR | 95% CI | |
| --- | --- | --- | --- | --- | --- | --- | --- | --- |
| Tsoi LC | mr_ivw_fe | LUCA | -0.0011 | 0.0223 | 0.9598 | 0.999 | 0.956 | 1.043 |
| Tsoi LC | mr_ivw_mre | LUCA | -0.0011 | 0.0329 | 0.9728 | 0.999 | 0.936 | 1.065 |
| Tsoi LC | mr_egger_regression | LUCA | 0.0299 | 0.0823 | 0.7179 | 1.030 | 0.877 | 1.211 |
| Tsoi LC | mr_weighted_median | LUCA | 0.0187 | 0.0366 | 0.6091 | 1.019 | 0.948 | 1.095 |
| Tsoi LC | mr_weighted_mode | LUCA | 0.0462 | 0.0677 | 0.4986 | 1.047 | 0.917 | 1.196 |
| Tsoi LC | mr_ivw_fe | LUSC | 0.0023 | 0.0345 | 0.9476 | 1.002 | 0.937 | 1.072 |
| Tsoi LC | mr_ivw_mre | LUSC | 0.0023 | 0.0426 | 0.9577 | 1.002 | 0.922 | 1.090 |
| Tsoi LC | mr_egger_regression | LUSC | 0.0398 | 0.1073 | 0.7127 | 1.041 | 0.843 | 1.284 |
| Tsoi LC | mr_weighted_median | LUSC | -0.0026 | 0.0535 | 0.9614 | 0.997 | 0.898 | 1.108 |
| Tsoi LC | mr_weighted_mode | LUSC | 0.0058 | 0.0798 | 0.9425 | 1.006 | 0.860 | 1.176 |
| Tsoi LC | mr_ivw_fe | LUAD | -0.0135 | 0.0340 | 0.6915 | 0.987 | 0.923 | 1.055 |
| Tsoi LC | mr_ivw_mre | LUAD | -0.0135 | 0.0488 | 0.7820 | 0.987 | 0.897 | 1.086 |
| Tsoi LC | mr_egger_regression | LUAD | -0.0019 | 0.1220 | 0.9877 | 0.998 | 0.786 | 1.268 |
| Tsoi LC | mr_weighted_median | LUAD | -0.0529 | 0.0522 | 0.3114 | 0.948 | 0.856 | 1.051 |
| Tsoi LC | mr_weighted_mode | LUAD | -0.0566 | 0.0877 | 0.5223 | 0.945 | 0.796 | 1.122 |
| FinnGen | mr_ivw_fe | LUCA | 0.0127 | 0.0404 | 0.7526 | 1.013 | 0.936 | 1.096 |
| FinnGen | mr_ivw_mre | LUCA | 0.0127 | 0.0558 | 0.8194 | 1.013 | 0.908 | 1.130 |
| FinnGen | mr_egger_regression | LUCA | -0.0106 | 0.1624 | 0.9497 | 0.989 | 0.720 | 1.360 |
| FinnGen | mr_weighted_median | LUCA | 0.0201 | 0.0583 | 0.7300 | 1.020 | 0.910 | 1.144 |
| FinnGen | mr_weighted_mode | LUCA | 0.0156 | 0.0734 | 0.8366 | 1.016 | 0.880 | 1.173 |
| FinnGen | mr_ivw_fe | LUSC | 0.0166 | 0.0611 | 0.7861 | 1.017 | 0.902 | 1.146 |
| FinnGen | mr_ivw_mre | LUSC | 0.0166 | 0.0747 | 0.8243 | 1.017 | 0.878 | 1.177 |
| FinnGen | mr_egger_regression | LUSC | -0.1202 | 0.2068 | 0.5769 | 0.887 | 0.591 | 1.330 |
| FinnGen | mr_weighted_median | LUSC | -0.0442 | 0.0838 | 0.5982 | 0.957 | 0.812 | 1.128 |
| FinnGen | mr_weighted_mode | LUSC | -0.0911 | 0.1119 | 0.4368 | 0.913 | 0.733 | 1.137 |
| FinnGen | mr_ivw_fe | LUAD | -0.0706 | 0.0619 | 0.2539 | 0.932 | 0.825 | 1.052 |
| FinnGen | mr_ivw_mre | LUAD | -0.0706 | 0.0965 | 0.4639 | 0.932 | 0.771 | 1.126 |
| FinnGen | mr_egger_regression | LUAD | 0.2579 | 0.2654 | 0.3597 | 1.294 | 0.769 | 2.177 |
| FinnGen | mr_weighted_median | LUAD | -0.0661 | 0.1035 | 0.5233 | 0.936 | 0.764 | 1.147 |
| FinnGen | mr_weighted_mode | LUAD | 0.1858 | 0.2096 | 0.3984 | 1.204 | 0.798 | 1.816 |

LUCA, lung cancer; LUSC, lung squamous cell cancer; LUAD, lung adenocarcinoma.

se, standard error; OR, odds ratio; CI, confidence interval.
