# Supplementary material for: Association between psoriasis and lung cancer: two-sample Mendelian randomization analyses
Source: BMC Pulm Med. 2023 Jan 5;23:4. doi: 10.1186/s12890-022-02297-0 (PMC9814449; doi:10.1186/s12890-022-02297-0)

SUPPLEMENTARY FIGURES

**Association between psoriasis and lung cancer: two-sample Mendelian randomization analyses**

Figure S1. Leave-one-out analysis for lung cancer, based on Tsoi LC.


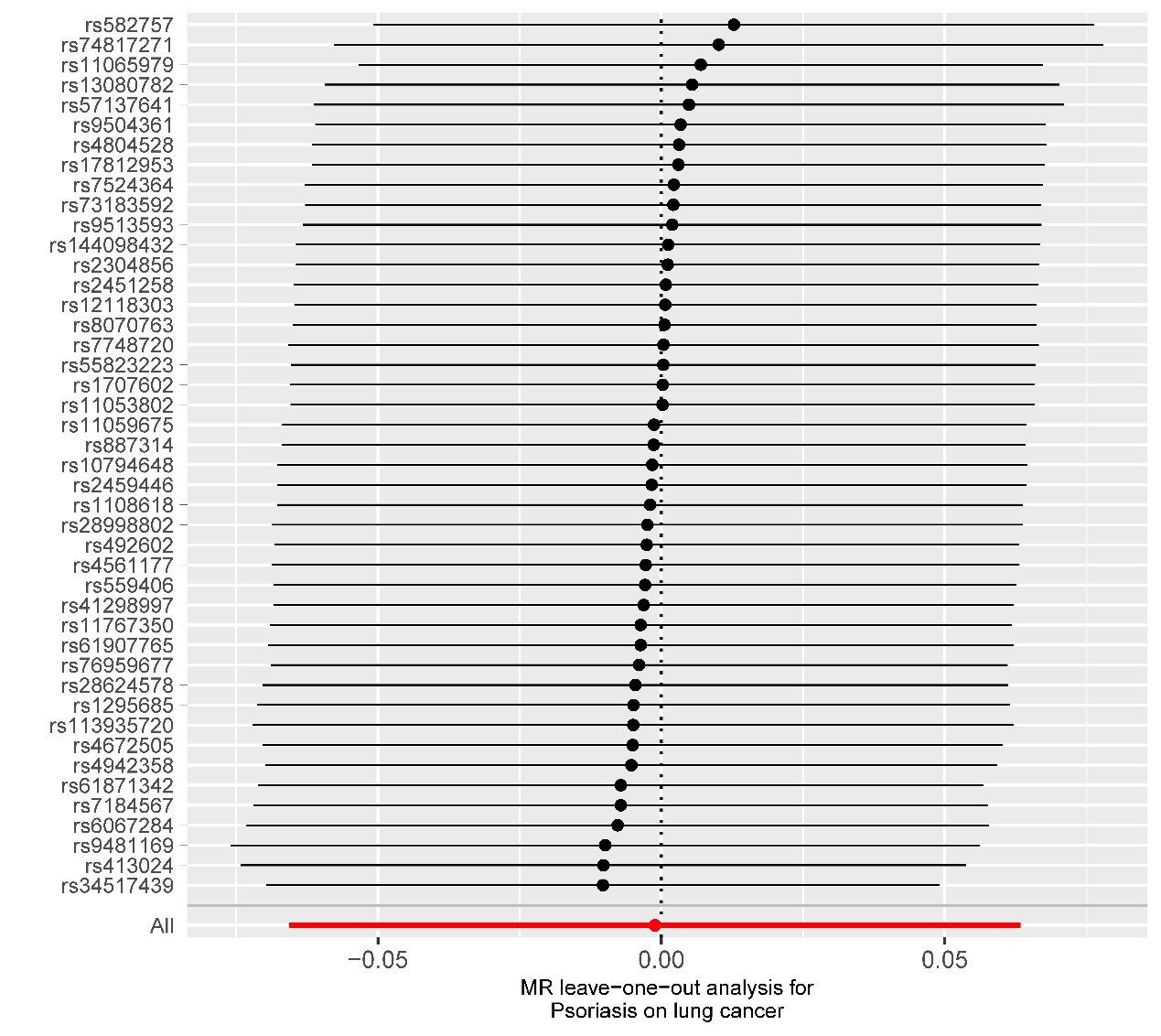


Figure S2. Leave-one-out analysis for squamous cell lung cancer, based on Tsoi LC.


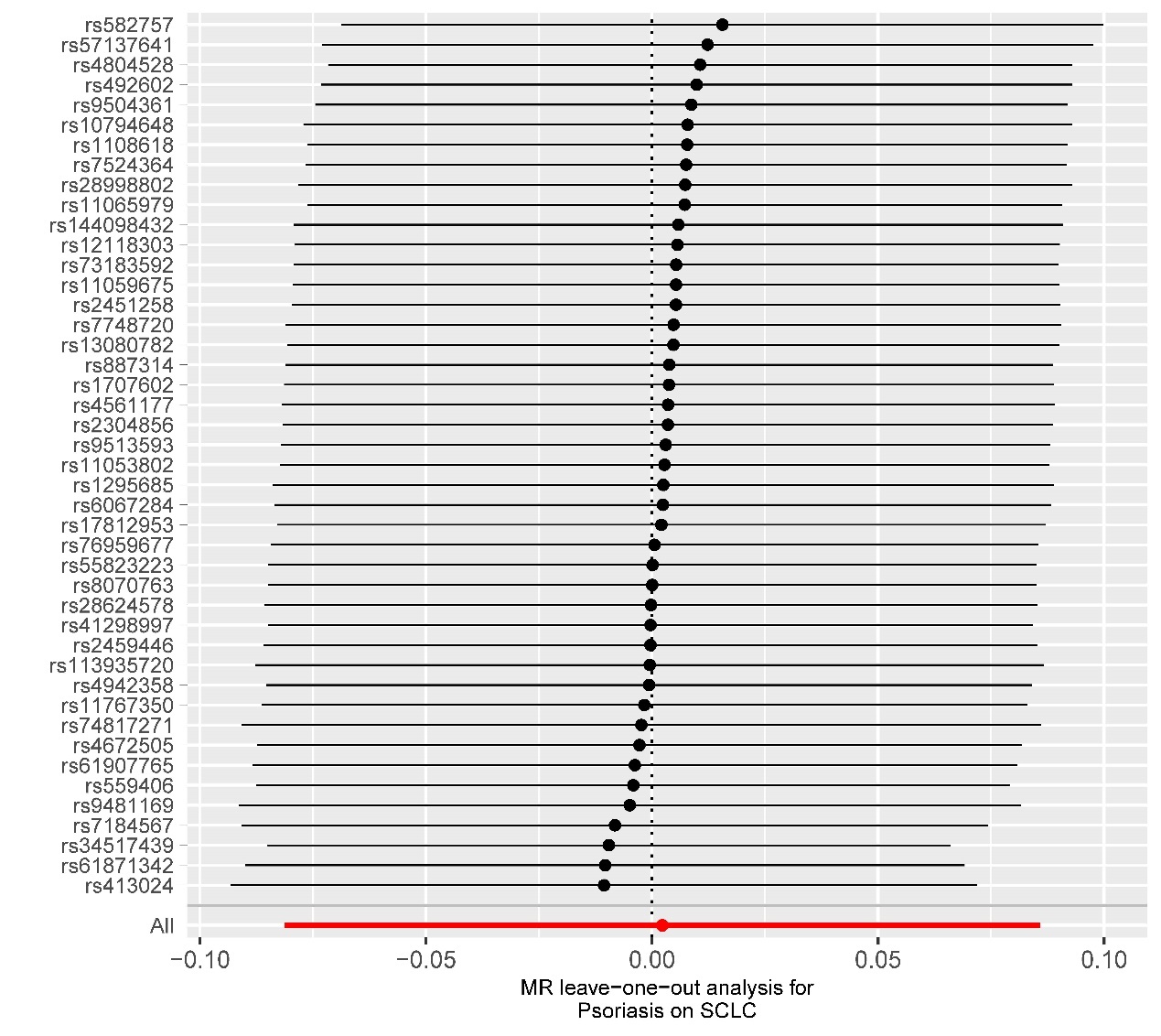


Figure S3. Leave-one-out analysis for lung adenocarcinoma, based on Tsoi LC.


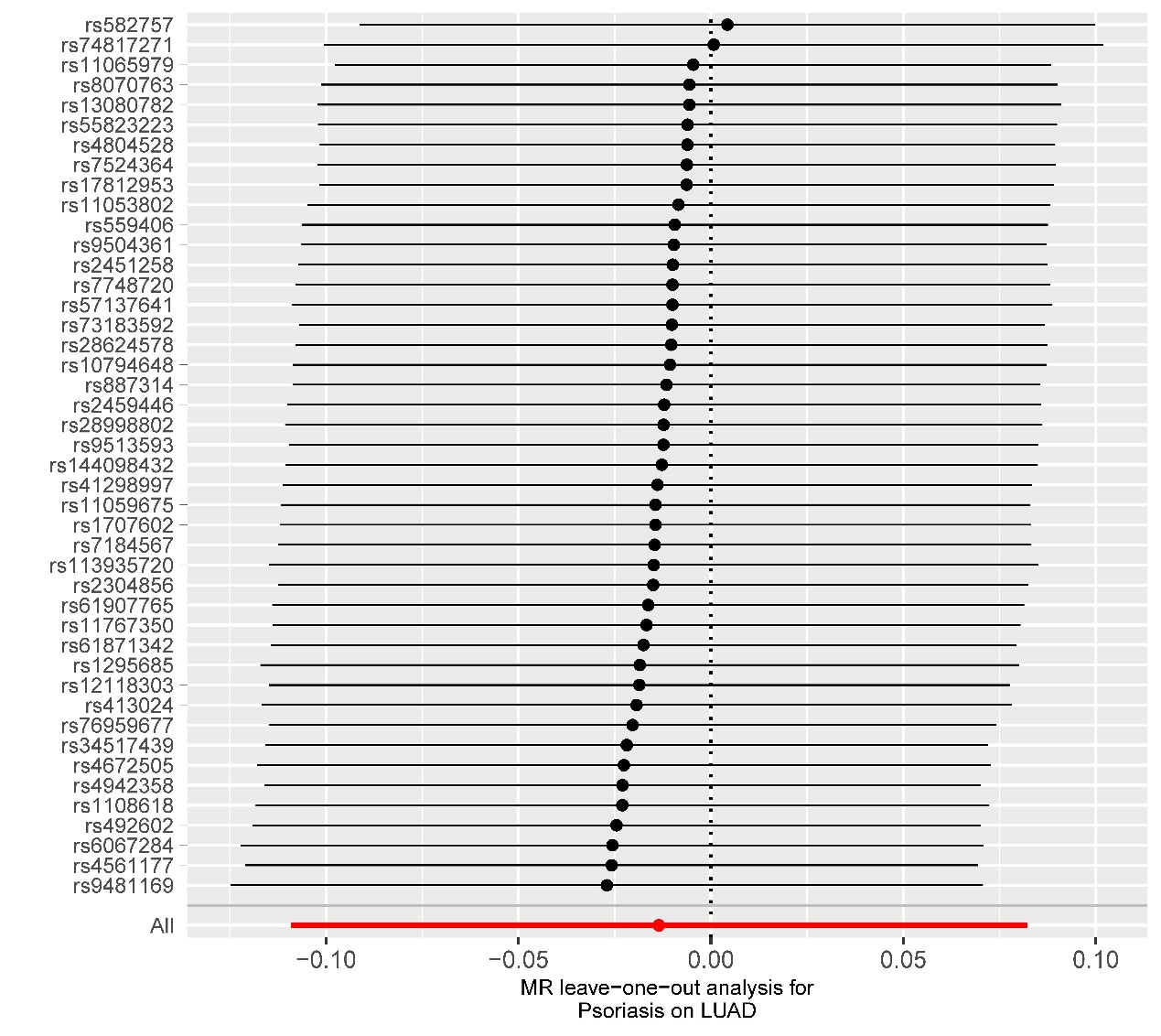


Figure S4. Leave-one-out analysis for lung cancer, based on FinnGen.


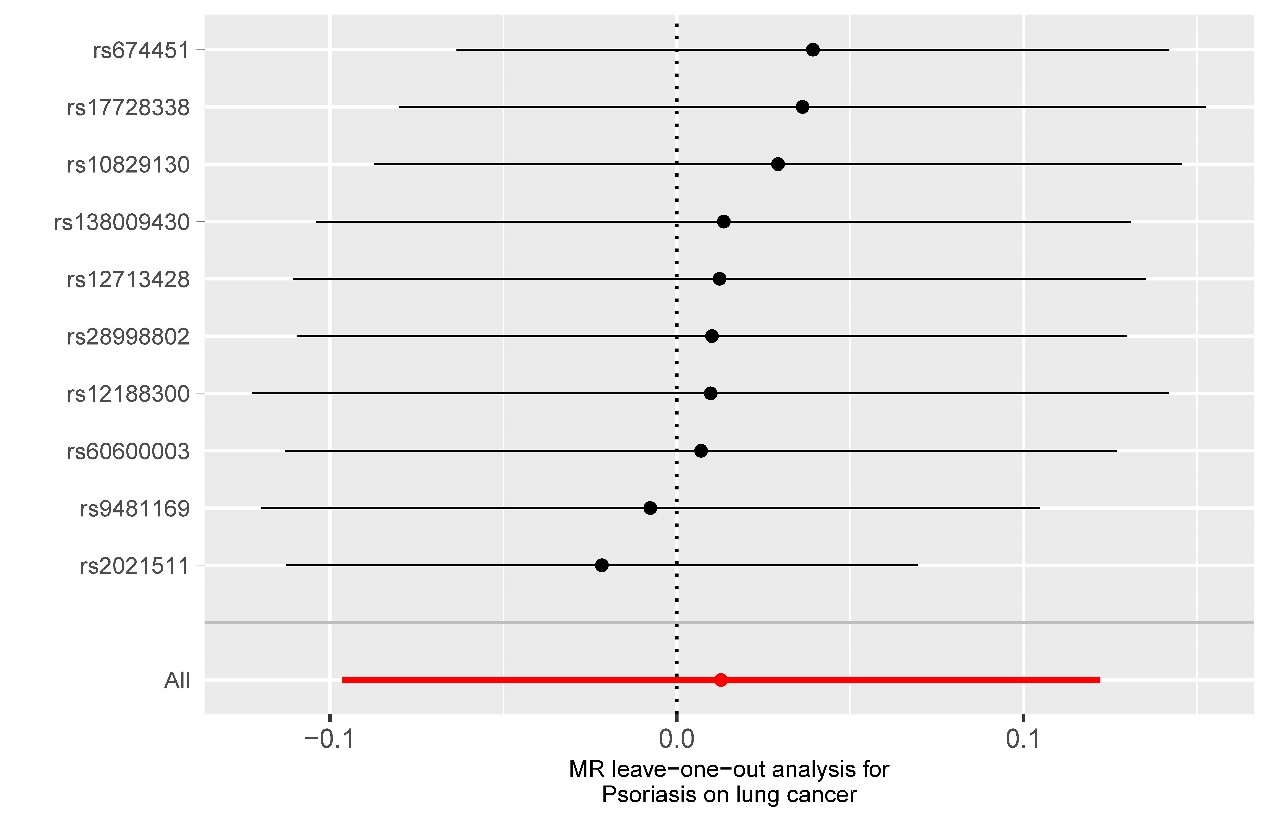


Figure S5. Leave-one-out analysis for squamous cell lung cancer, based on FinnGen.


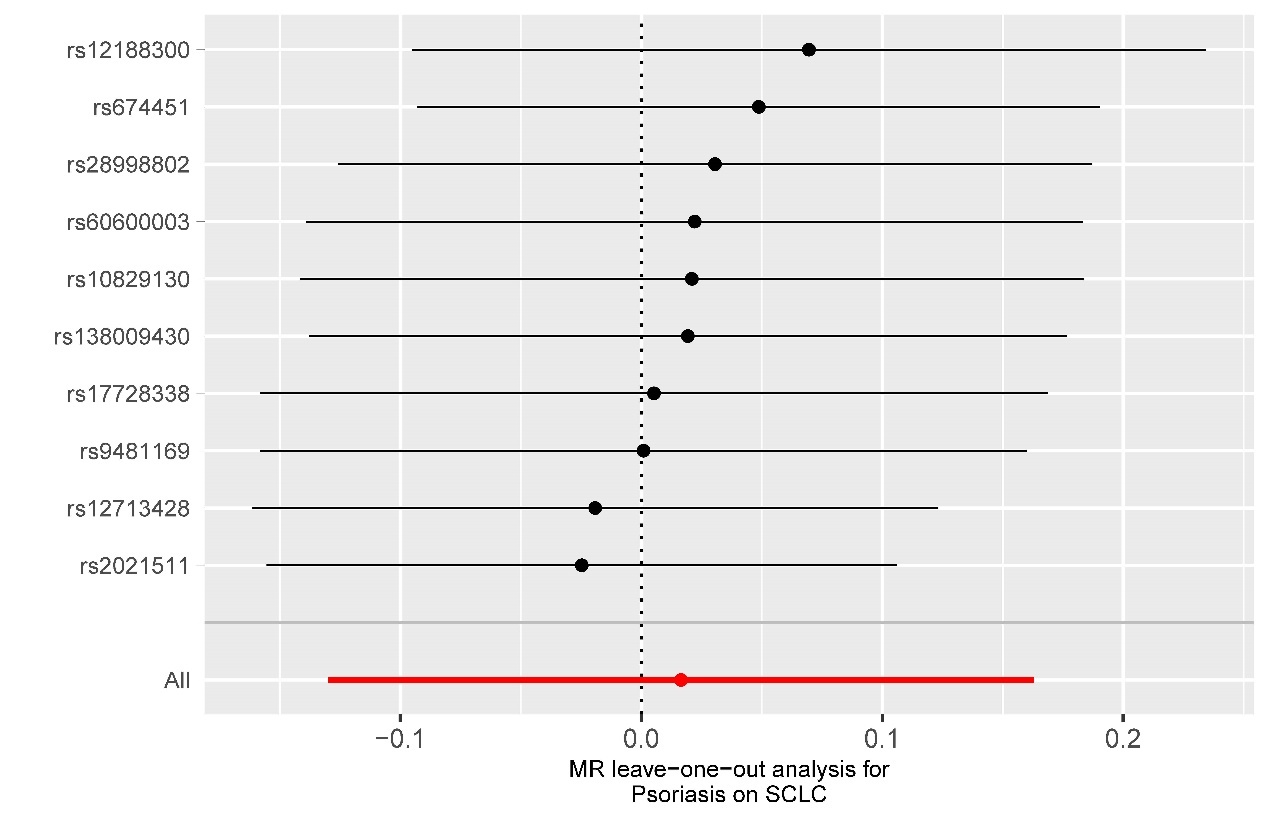


Figure S6. Leave-one-out analysis for lung adenocarcinoma, based on FinnGen.


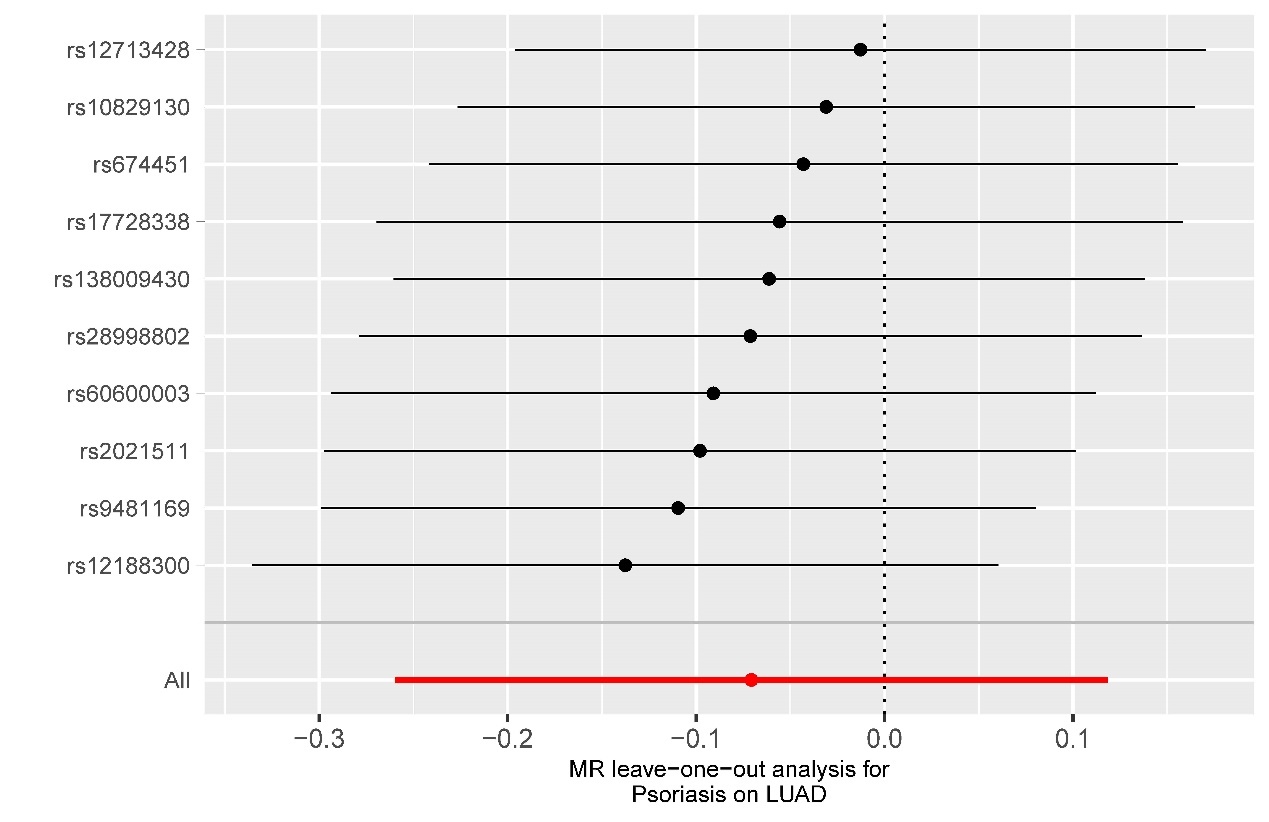

Supplement: Supplementary file 2 — Additional file 2. Figure S1 Leave-one-out analysis for lung cancer based on GWAS of Tsoi LC. Figure S2 Leave-one-out analysis for squamous cell lung cancer based on GWAS of Tsoi LC. Figure S3 Leave-one-out analysis for lung adenocarcinoma based on GWAS of Tsoi LC. Figure S4 Leave-one-out analysis for lung cancer based on GWAS of FinnGen. Figure S5 Leave-one-out analysis for squamous cell lung cancer based on GWAS of FinnGen. Figure S6 Leave-one-out analysis for lung adenocarcinoma based on GWAS of FinnGen. [file 12890_2022_2297_MOESM2_ESM.docx]
